# Screening for COVID-19: Patient factors predicting positive PCR test

**DOI:** 10.1017/ice.2020.249

**Published:** 2020-05-19

**Authors:** Douglas W. Challener, Gregory J. Challener, Vanessa J. Gow-Lee, Madiha Fida, Aditya S. Shah, John C. O’Horo

**Affiliations:** 1 Division of Infectious Diseases, Mayo Clinic, Rochester, Minnesota; 2 Department of Internal Medicine, Mayo Clinic, Rochester, Minnesota; 3 Division of Pulmonary and Critical Care Medicine, Rochester, Minnesota

## Abstract

To inform the efficient allocation of testing resources, we evaluated the characteristics of those tested for COVID-19 to determine predictors of a positive test. Recent travel and exposure to a confirmed case were both highly predictive of positive testing. Symptom-based screening strategies alone may be inadequate to control the ongoing pandemic.

SARS-CoV-2, the novel coronavirus causing COVID-19, was isolated in patients from Wuhan, China, in December 2019 and sparked a global pandemic in early 2020.^1,2^ Symptom-based and exposure-based screening was recommended by the US Centers for Disease Control (CDC) in late February 2020 as the virus began to spread throughout the United States. Unfortunately, current evidence suggests that symptom-based screening programs are likely to miss a large proportion of infected cases.^3–5^

The containment of an infectious disease of large public health consequence relies on case identification, contact tracing, and isolation. At Mayo Clinic in Rochester, Minnesota, we developed a polymerase chain reaction (PCR) assay^6^ for SARS-CoV-2 and deployed a drive-through specimen collection site on March 12, 2020, that was modelled after similar interventions in South Korea and Washington state.^7^ To inform efficient allocation of limited testing resources, we sought to identify patient characteristics most predictive of a positive test.

## Methods

At the Mayo Clinic in Rochester, Minnesota, we began screening patients for COVID-19 on a large scale on March 12, 2020, after Minnesota’s first case was reported on March 10, 2020. Patients who were screened were given a standardized questionnaire by a nurse prior to testing. This questionnaire included questions about patient symptoms such as fever (subjective or objective), cough, shortness of breath, and medical comorbidities. The patients were also asked about recent travel as well as exposure to laboratory-confirmed cases of COVID-19.

We examined the medical records of patients with the first 48 positive tests and a selection of 98 patients with negative tests. The COVID-19–negative patients were selected in a random fashion by matching age (±5 years), sex, collection date, and testing location (Minnesota, Wisconsin, or Arizona) with the positive patients. Each positive patient had at least a single negative control. All patients were screened between March 12 and March 26, 2020. The chart of each patient was then manually abstracted by a physician to identify patient characteristics, symptoms, and potential exposures identified by the nurse triage line as reasons to recommend screening prior to each individual’s test date. Travel to a major metropolitan area was also recorded. Study data were collected and managed using REDCap electronic data capture tools hosted at the Mayo Clinic.^7,8^ Descriptive statistics, *t* tests, and logistic regression analysis were performed using JMP version 14 software (SAS Institute, Cary, NC). Our institutional review board approved this study.

## Results

The average age in the cohort was ~46 years, with slightly more men than women (Table 1). Due to the matching strategy for negative controls, there was no statistically significant difference between the 2 groups. Patients with both negative and positive tests had high rates of fever and cough, which likely led to the initial decision to screen them. Overall, the cohort had few medical comorbidities.

**Table 1.** Characteristics of Patients Who Were Tested for COVID-19

Characteristic	Positive Test (n=48), No. (%)	Negative Test (n=98), No. (%)	*P* Value
Age, mean y (SD)	45.9 (19.0)	46.0 (16.0)	.98
Sex, male	26 (54)	61 (62)	.37
Healthcare worker	12 (25)	19 (20)	.94
Iatrogenic immunocompromise	2 (4.4)	5 (5.1)	1
Chronic pulmonary disease (asthma, COPD, or ILD)	6 (13)	30 (31)	.02
Congestive heart failure	1 (2)	4 (4)	.57
End-stage renal disease	0 (0)	1 (1)	.99
End-stage liver disease	0 (0)	0 (0)	1
Close exposure to lab-confirmed case of COVID-19	13 (29.5)	5 (5.6)	<.01
Recent travel to major metropolitan area	33 (73)	38 (44)	<.01
Cough	42 (93)	92 (94)	.90
Fever	36 (80)	83 (86)	.33

Note. COVID-19, novel coronavirus 2019; SD, standard deviation; COPD, chronic obstructive pulmonary disease; ILD, interstitial lung disease.

The largest differentiating factors between the patients with positive and negative tests were exposures. Patients with positive tests were significantly more likely to have travelled to a major metropolitan area within the preceding 2 weeks or to have come into contact with a person with laboratory-confirmed COVID-19. In a multivariable logistic regression model predicting a positive test adjusted for these 2 factors, close contact with a confirmed case increased the odds of a positive test by 17 times (95% CI, 4.6–88.4), and recent travel increased the odds of a positive test by 4.7 times (95% CI, 1.9-12.7).

## Discussion

The selection of patients for SARS-CoV-2 screening remains challenging. Many factors influence the decisions on which patients to screen, including testing resources, test characteristics (sensitivity and specificity), and local disease prevalence. The challenge in determining the appropriate patients to screen has been apparent; the CDC has revised its guidance several times. This study investigates the results of testing ambulatory patients in a relatively low prevalence area in early March 2020 and suggests that exposure to the disease is more predictive of a positive test than any examined symptom.

This retrospective analysis of the initial phase of our screening for COVID-19 had several strengths. A rigorous physician review of each medical record helped ensure accurate capture of patient information. Additionally, the short study period helped limit any major local factors that could have affected the results, such as changing screening guidelines or increasing community prevalence. Furthermore, all the tests were collected, transported, and analyzed within the same internal institutional laboratory process.

This study also had several limitations. First, this was a retrospective analysis; thus, it may have suffered from selection bias affecting the participants. To help avert this bias, our negative controls were matched for sex, age, date, and state of collection. In addition, very few asymptomatic patients were screened during this time, making it difficult to assess the predictive value of fever or cough. Moreover, at the time of this study, local disease prevalence was relatively low, thereby limiting the applicability of the findings to higher prevalence areas.

Although testing for COVID-19 remains supply constrained, strategies are needed to best utilize testing resources. Identifying patient factors that are strongly associated with positive results may help to identify those patients best suited for testing. In this analysis, exposure to confirmed SARS-CoV-2 and recent travel were both significantly more predictive of a positive test than the presence of any symptoms. In the effort to contain the pandemic, there may be a role for testing patients with these risk factors regardless of symptom presence.
